# Supplementary material for: Immunogenicity and structure of a stabilized HIV-1 clade C Env from a pediatric elite neutralizer in complex with an autologous bnAb
Source: Front Immunol. 2026 Oct 21;17:1919627. doi: 10.3389/fimmu.2026.1919627 (PMC13731313; doi:10.3389/fimmu.2026.1919627)
Supplement: Supplementary file 1 [file DataSheet1.pdf]

```
                ....|....| ....|....| ....|....| ....|....| ....|....| ....|....| ....|....| ....|....|
                        10         20         30         40         50         60         70         80
HXB2            MRVKEKYQHL WRWGWRWGTM LLGMLMICSA TEKLWVTVYY GVPVWKEATT TLFCASDAKA YDTEVHNVWA THACVPTDPN
330.16.E6       MKVRGTLRNY RQW-WIWGVL GFWMLMICNV EGDSWVTVYY GVPVWKEAKT TLFCASDAKA YDKEVHNVWA THACVPTDPS
330.NFL         ---MGSLQPL ATL-YLLGML ----VASVLA AGNLWVTVYY GVPVWKDAET TLFCASDAKA YDKEKHNVWA THACVPTDPS
330.SOSIP       ---MGSLQPL ATL-YLLGML ----VASVLA AGNLWVTVYY GVPVWKDAET TLFCASDAKA YDKEKHNVWA THACVPTDPS
Consensus           . :        :  * :      :    .    . ****** ******:* * ********** **.* ***** *********.

                ....|....| ....|....| ....|....| ....|....| ....|....| ....|....| ....|....| ....|....|
                        90        100        110        120        130        140        150        160
HXB2            PQEVVLVNVT ENFNMWKNDM VEQMHEDIIS LWDQSLKPCV KLTPLCVSLK CTDLKNDTNT NSSSGRMIME KGEIKNCSFN
330.16.E6       PQEMVLENVT ENFNMWKNDM VDQMQEDVIS LWDQSLKPCV KLTPLCVTLI CANVNSNSST TLN---ETHD GSELKNCSFN
330.NFL         PQEMVLENVT ENFNMWKNDM VDQMQTDVIS LWDQSLKPCV KLTPLCVTLI CANVNSNSST TLN---ETHD GSELKNCSFN
330.SOSIP       PQEMVLENVT ENFNMWKNDM VDQMQTDVIS LWDQSLKPCV KLIPLWVTLI CANGNSKSST TLN---ETHD GSELKNCSFN
Consensus       ***:** *** ********** *:**: *:** ********** ** ** *:*   *:: :..:.* . .      :  .*:******

                ....|....| ....|....| ....|....| ....|....| ....|....| ....|....| ....|....| ....|....|
                       170        180        190        200        210        220        230        240
HXB2            ISTSIRGKVQ KEYAFFYKLD IIPIDN---D TTSYKLTSCN TSVITQACPK VSFEPIPIHY CAPAGFAILK CNNKTFNGTG
330.16.E6       ATTEIRDRKQ TVHALFYRLD ILPLDEKNKN SSKYRLINCN TSAITQACPK VTFDPIPIHY CAPAGYAILK CNNKTFNGTG
330.NFL         ATTELRDRKQ KVHALFYRLD ILPLDEKNKN SSKYRLINCN TSACTQACPK VTFDPIPIHY CAPAGYAILK CNNKTFNGTG
330.SOSIP       GTTELRDRKQ KVHALFYRLD ILPLDEKNKN SSKYRLINCN TSACTQACPK VTFDPIPIHY CAPAGYAILK CNNKTFNGTG
Consensus        :*.:*.: * . :*:**:** *:*:*:   : ::.*:* .** **. ****** *:*:****** *****:**** **********

                ....|....| ....|....| ....|....| ....|....| ....|....| ....|....| ....|....| ....|....|
                       250        260        270        280        290        300        310        320
HXB2            PCTNVSTVQC THGIRPVVST QLLLNGSLAE EEVVIRSVNF TDNAKTIIVQ LNTSVEINCT RPNNNTRKRI RIQRGPGRAF
330.16.E6       PCHNVSTVQC THGIKPVVST QLLLNGSLAE GEIIIRSENL TNNVKTIIVH LNQSVEIVCT RPNNNTRKSI RIGPGQ-TFY
330.NFL         PCHNVSTVQC THGIKPVVST QLLLNGSLAE GEIIIRSENL TNNVKTIIVH LNQSVEIVCT RPNNYTRKSI RIGPGQ-TFY
330.SOSIP       PCHNVSTVQC THGIKPVVST QLLLNGSLAE GEIIIRSENL TNNVKTIIVH LNQSVEIVCT RPNNYTRKSI RIGPGQ-TFY
Consensus       ** ******* ****:***** **********  *::*** *: *:*.*****: ** **** ** **** *** * **  *    :

                ....|....| ....|....| ....|....| ....|....| ....|....| ....|....| ....|....| ....|....|
                       330        340        350        360        370        380        390        400
HXB2            VTIGKIGNMR QAHCNISRAK WNNTLKQIAS KLREQFGNNK TIIFKQSSGG DPEIVTHSFN CGGEFFYCNS TQLFNSTWFN
330.16.E6       AT-DIIGDIR QAHCNISEKK WHETLKGVGK KLAEHFPN-K TIKFTSSSGG DLEITMHSFN CRGEFFYCNT SGLFNSTYNG
330.NFL         ATGDIIGDIR QAHCNISEKK WHETLKGVGK KLAEHFPN-K TIKFTSSSGG DLEITMHSFN CRGEFFYCNT SGLFNSTYNG
330.SOSIP       ATGDIIGDIR QAHCNISEKK WHETLKGVGK KLAEHFPN-K TIKFTSSSGG DLEITMHSFN CRGEFFYCNT SGLFNSTYNG
Consensus       .* . **::* *******. * *::*** :.. ** *:* * * ** *..**** * **. **** * *******: : *****: .

                ....|....| ....|....| ....|....| ....|....| ....|....| ....|....| ....|....| ....|....|
                       410        420        430        440        450        460        470        480
HXB2            STWSTEGSNN TEGS-----D TITLPCRIKQ IINMWQKVGK AMYAPPISGQ IRCSSNITGL LLTRDGGNSN --NESEIFRP
330.16.E6       TSGLFNSTYN GTYSHDGVNS TITLPCRIKQ IINMWQEVGR AMYAPPIEGN ITCKSNITGI LLVRDGGDNN -QSDTETFRP
330.NFL         TSGLFNSTYN ---------S TITLPCRIKQ IINMWQRVGQ CMYAPPIEGN ITCKSNITGI LLVRDGGDNN -QSDTETFRP
330.SOSIP       TSGLFNSTYN ---------S TITLPCRIKQ IINMWQRVGQ CMYAPPIEGN ITCKSNITGI LLVRDGGDNN HQSDTETFRP
Consensus       ::   :.: *          . ********** ******.**: .******.*: * *.*****: **.****:.*    .::* ***

                ....|....| ....|....| ....|....| ....|....| ....|....| ....|....| ....|....| ....|....|
                       490        500        510        520        530        540        550        560
HXB2            GGGDMRDNWR SELYKYKVVK IEPLGVAPTK AKRRVVQ--- ---REKRAVG IGALFLGFLG AAGSTMGAAS MTLTVQARQL
330.16.E6       GGGDMRDNWR SELYKYKVVE IKPLGVAPTA AKRRVVE--- ---RGKRAVG IGAVFLGFLG AAGSTMGAAS ITLTVQARQL
330.NFL         GGGDMRDNWR SELYKYKVVE IKPLGVAPTR AKRRVVEGGG GSGGGGSAVG IGAVRRGFLG AAGSTMGAAS ITLTVQARQL
330.SOSIP       GGGDMRDNWR SELYKYKVVE IKPLGVAPTR CKRRVVE--- -RRRRRRAVG IGAVSLGFLG AAGSTMGAAS MTLTVQARNL
Consensus       ********** *********: *:*******  .*****:            *** ***:  **** ********** :*******:*

                ....|....| ....|....| ....|....| ....|....| ....|....| ....|....| ....|....| ....|....|
                       570        580        590        600        610        620        630        640
HXB2            LSGIVQQQNN LLRAIEAQQH LLQLTVWGIK QLQARILAVE RYLKDQQLLG IWGCSGKLIC TTAVPWNASW SNKSLEQIWN
330.16.E6       LSGIVQQQSN LLRAIEAQQH LLQLTVWGIK QLQTRVLAIE RYLKDQQLLG LWGCSGKLIC TTAVPWNSSW SNKSQEDIWN
330.NFL         LSGIVQQQSN LPRAPEAQQH LLQLGVWGIK QLQTRVLAIE RYLKDQQLLG LWGCSGKLIC TTAVPWNSSW SNKSQEDIWN
330.SOSIP       LSGIVQQQSN LLRAPECQQH LLQDTHWGIK QLQTRVLAIE HYLKDQQLLG LWGCSGKLIC CTAVPWNSSW SNKSQEDIWN
Consensus       ********.* * ** *.*** ***    **** ***:*:**:* :********* :*********  ******:** **** *:***

                ....|....| ....|....| ....|....| ....|....|
                       650        660        670        680
HXB2            HTTWMEWDRE INNYTSLIHS LIEESQNQQE KNEQELLELD
330.16.E6       NMTWMQWDRE INNYTNTIYQ LLEESQNQQE KNEKDLLALD
330.NFL         NMTWMQWDRE IGNYTNTIYQ LLEESQNQQE KNEVDLLALD
330.SOSIP       NMTWMQWDRE IGNYTNTIYQ LLEESQNQQE KNEVDLLALD
Consensus       : ***:**** *.***. *:. *:******** *** :** **
```

Figure S1. Sequence alignment of 330 gp140 SOSIP and NFL amino acid sequences to the HXB2 reference coordinates.

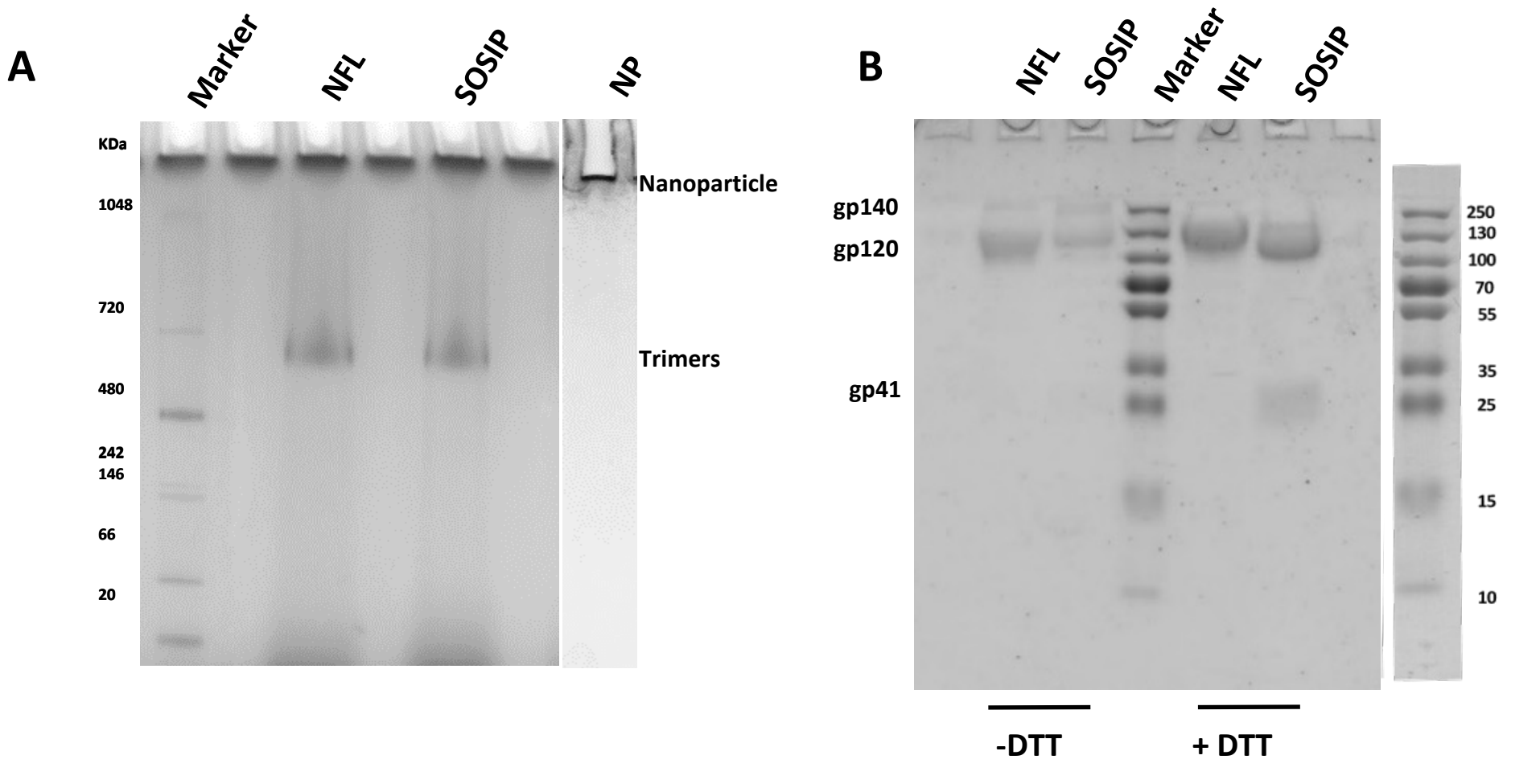

Figure S2. Biochemical characterization of 330 gp140 Env trimers and ferritin nanoparticles. (A) Blue Native-PAGE analysis of PGT145 purified 330 gp140 SOSIP and NFL trimers, along with 330 ferritin NPs, stained with Coomassie blue. The soluble Env trimers migrated as discrete high-molecular-weight bands consistent with intact trimeric species, whereas the ferritin NPs remained predominantly at the top of the gel because of their large molecular size. **(B)** SDS-PAGE analysis of purified 330 gp140 Env trimers under reducing and non-reducing conditions. Under reducing conditions (+DTT), the Env proteins migrated predominantly as monomeric gp120 and gp41 species, indicating the purity and integrity of the trimer preparations.

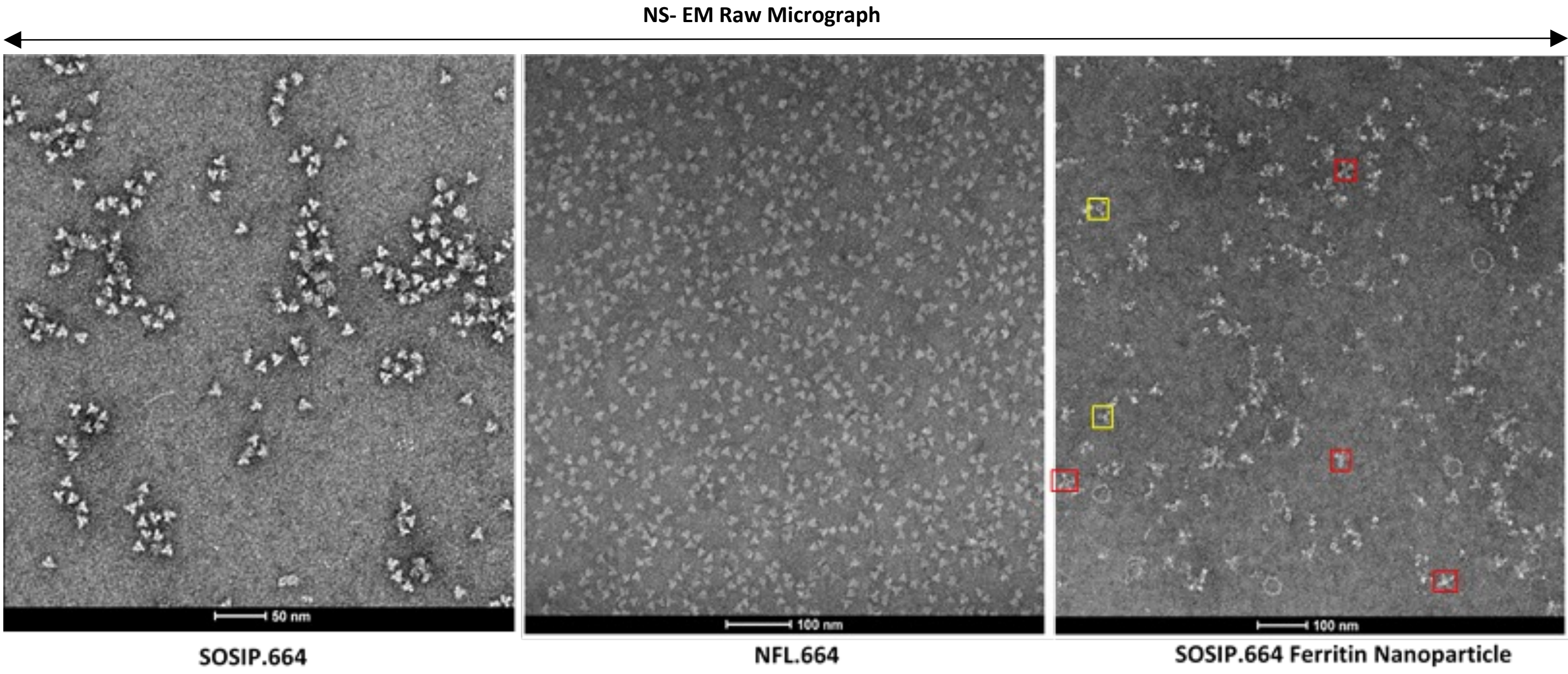

Figure S3. Negative staining electron microscopy raw micrographs. (A) Representative negative-stain TEM (nsTEM) raw micrographs of 330 SOSIP env trimers at 50 nm scale. **(B)** Raw micrograph representing 330 NFL env trimers at 100 nm scale. **(C)** Representative micrographs of self-assembling H. pylori ferritin nanoparticles presenting the SOSIP Env trimers, red indicating four or more trimers presentation, while yellow indicates up to two Env presentations by ferritin particles.

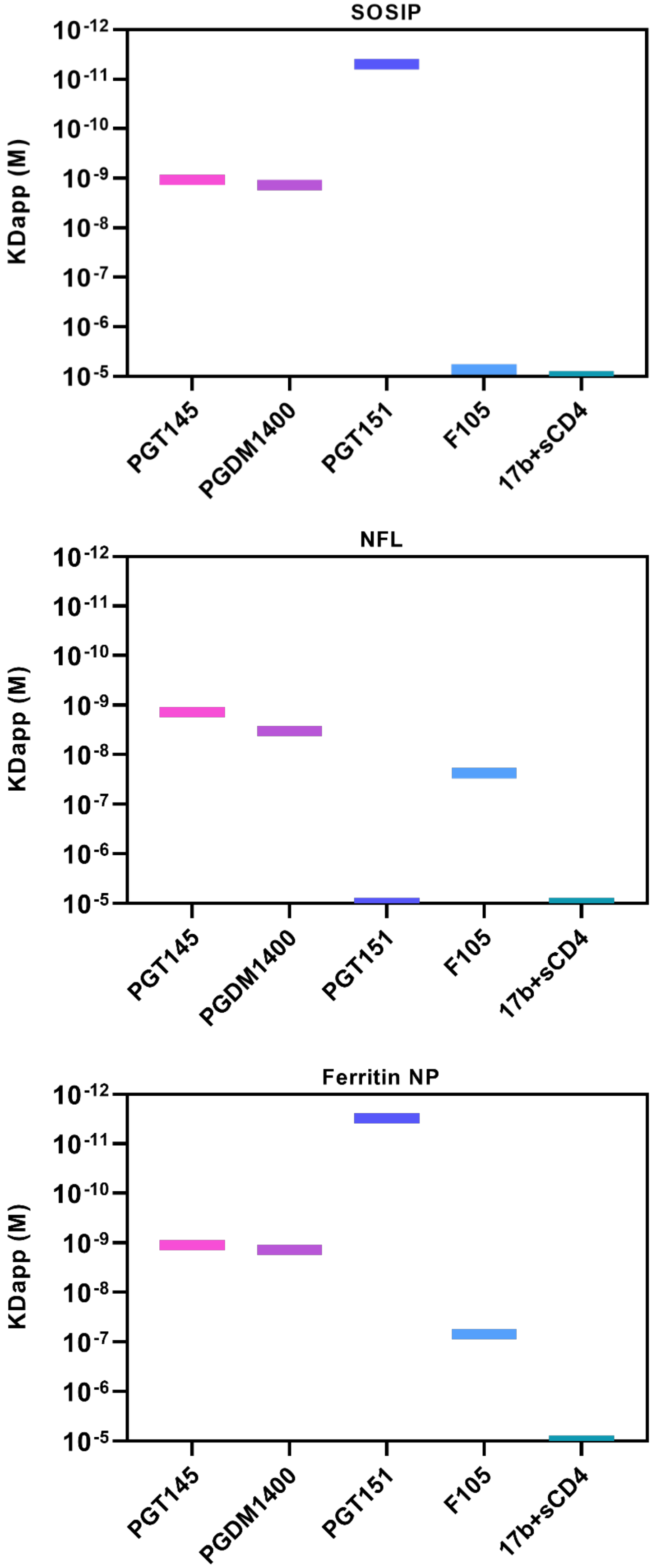

Figure S4. BLI-Octet analysis of antibody binding to 330 gp140 Env trimers and ferritin nanoparticles. Binding affinities of 330 SOSIP, NFL, and ferritin NPs to bnAbs PGT145, PGDM1400, and PGT151 and non-nAbs F105 and 17b in the presence of sCD4 were determined by biolayer interferometry (BLI). Apparent KD values (K_D^{app}) are shown on a logarithmic scale in molar (M) units. The KD values were derived using 1:1 global fitting.

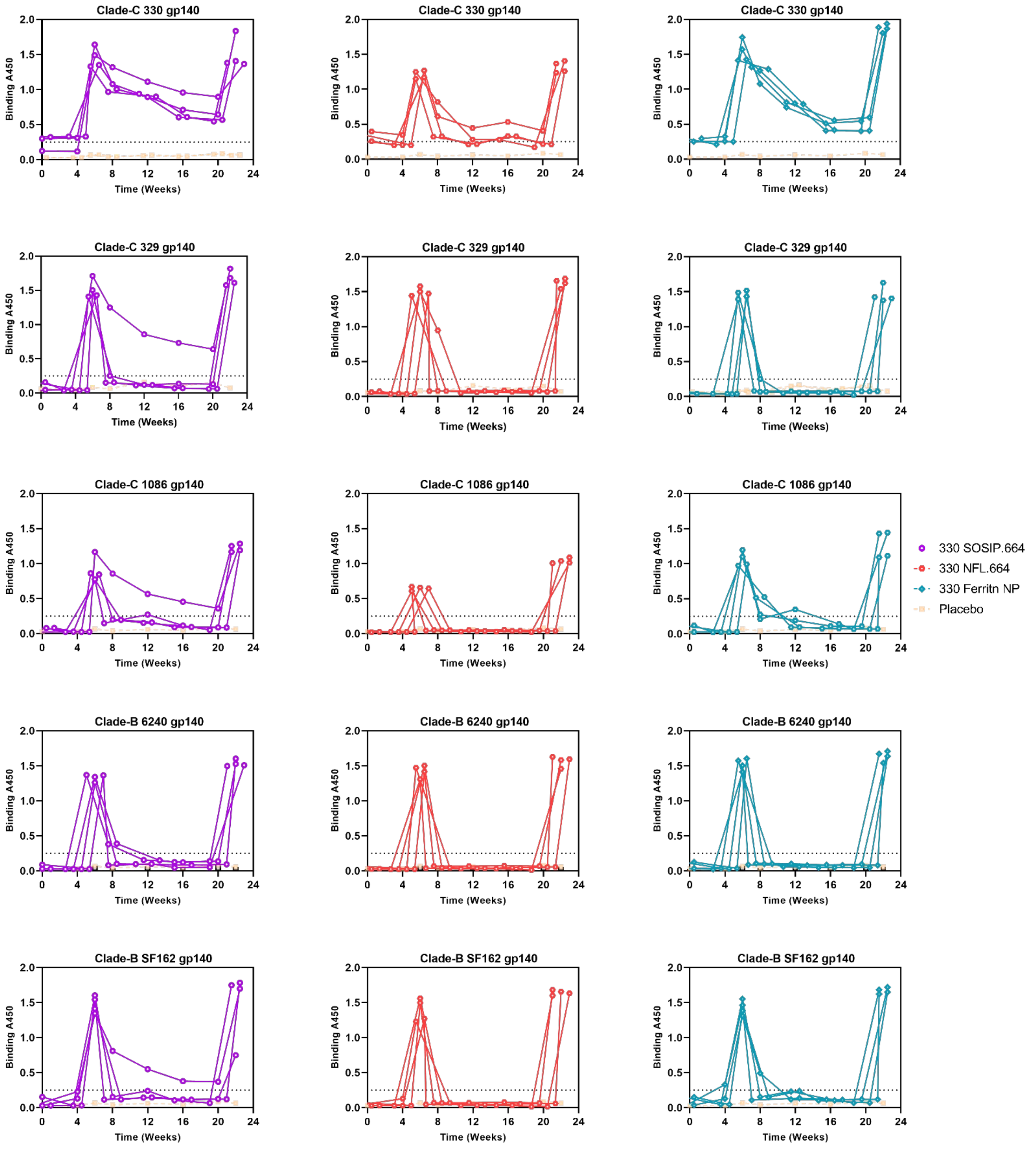

Figure S5. Longitudinal binding endpoint titers of individual animals in each group. Serum antibody endpoint titers were determined longitudinally by ELISA against the clade C autologous Env 330 and heterologous HIV-1 gp140 Env proteins, including clade C Envs 329 and 1086 and clade B Envs SF162 and 6240. Individual animal endpoint titers are shown for each time point.

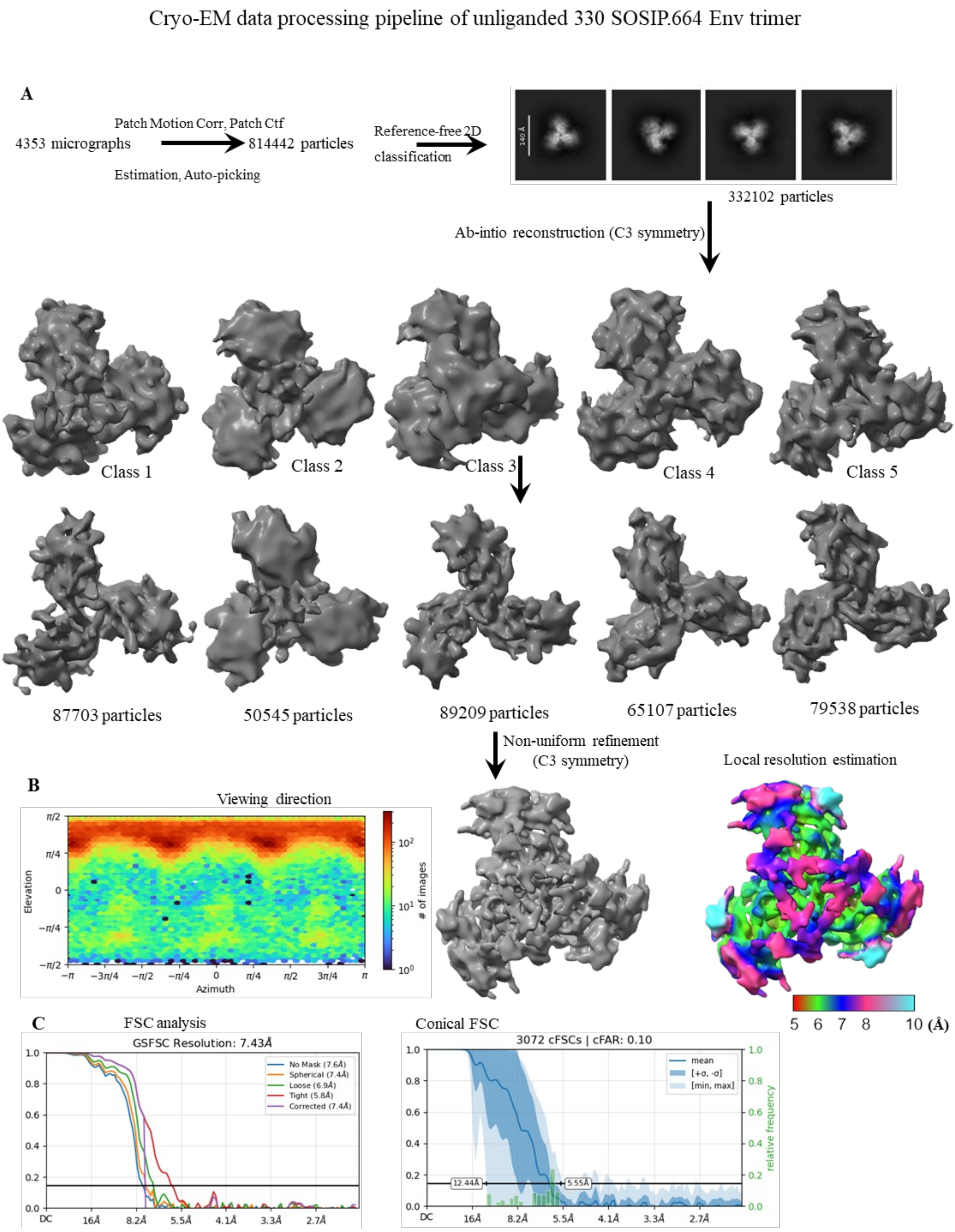

Figure S6: Cryo-EM analysis of unliganded 330 SOSIP.664 Env trimer (A) Pipeline of Cryo-EM data processing using single particle cryo-EM and 3D classification of 330 SOSIP.664 Env trimer. **(B)** The viewing-direction particle distribution for Class 04 reveals diverse particle orientations in the dataset. **(C)** Gold standard Fourier Shell Correlation (FSC) curve of the cryo-EM map at 7.43 Å of 330 SOSIP.664 Env trimer.

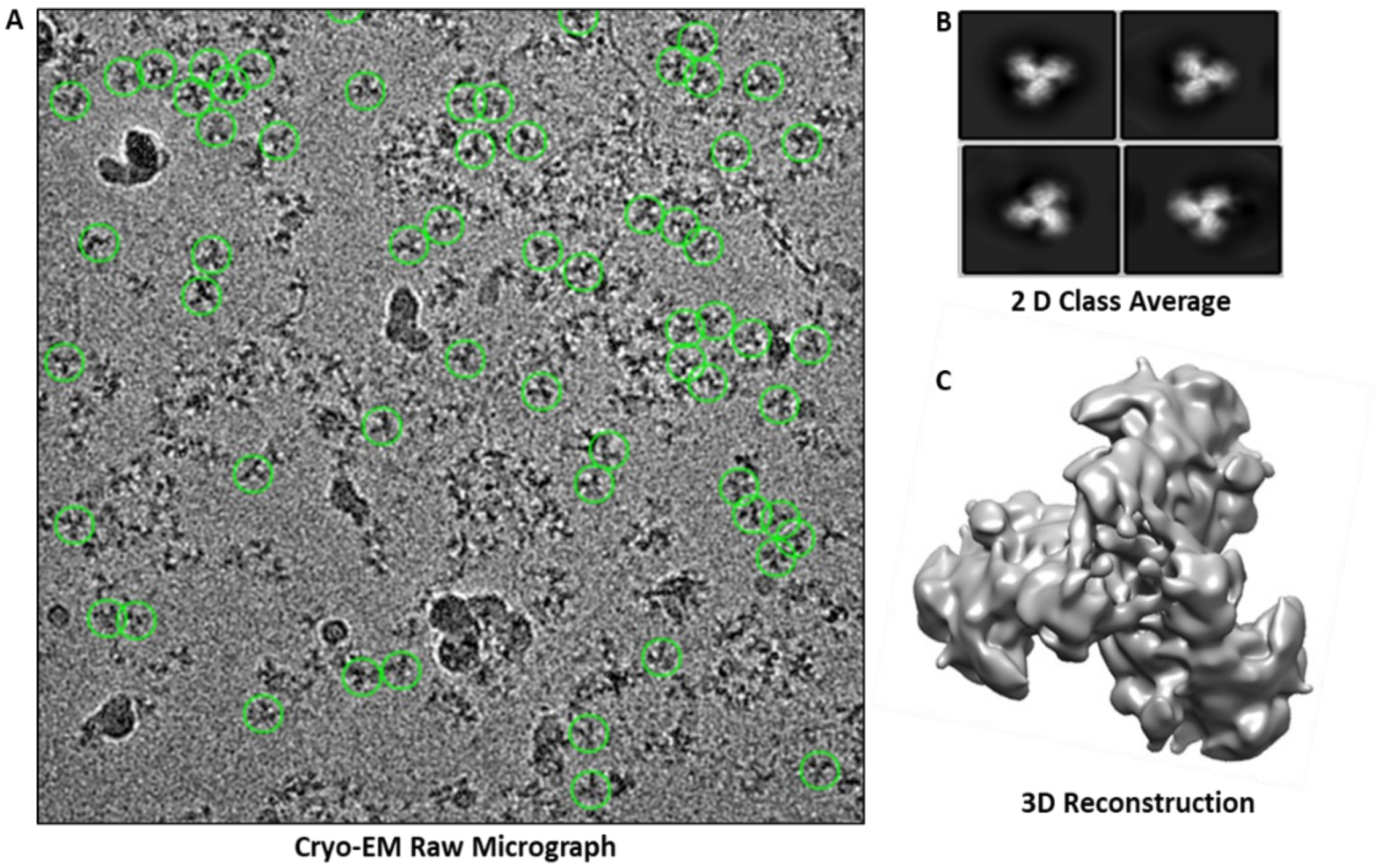

Figure S7: Single particle cryo-Electron Microscopy (cryo-EM) of native like 330 SOSIP gp140 env trimers (A) Raw Cryo-EM micrograph representing 330 SOSIP env trimers **(B)** Cryo-EM 2-D class averages of SOSIP gp140 env trimers **(C)** 3D reconstruction of soluble gp140 trimers in prefusion closed conformation.

Cryo-EM data processing pipeline of 330 Env complexed with 44m bnAb

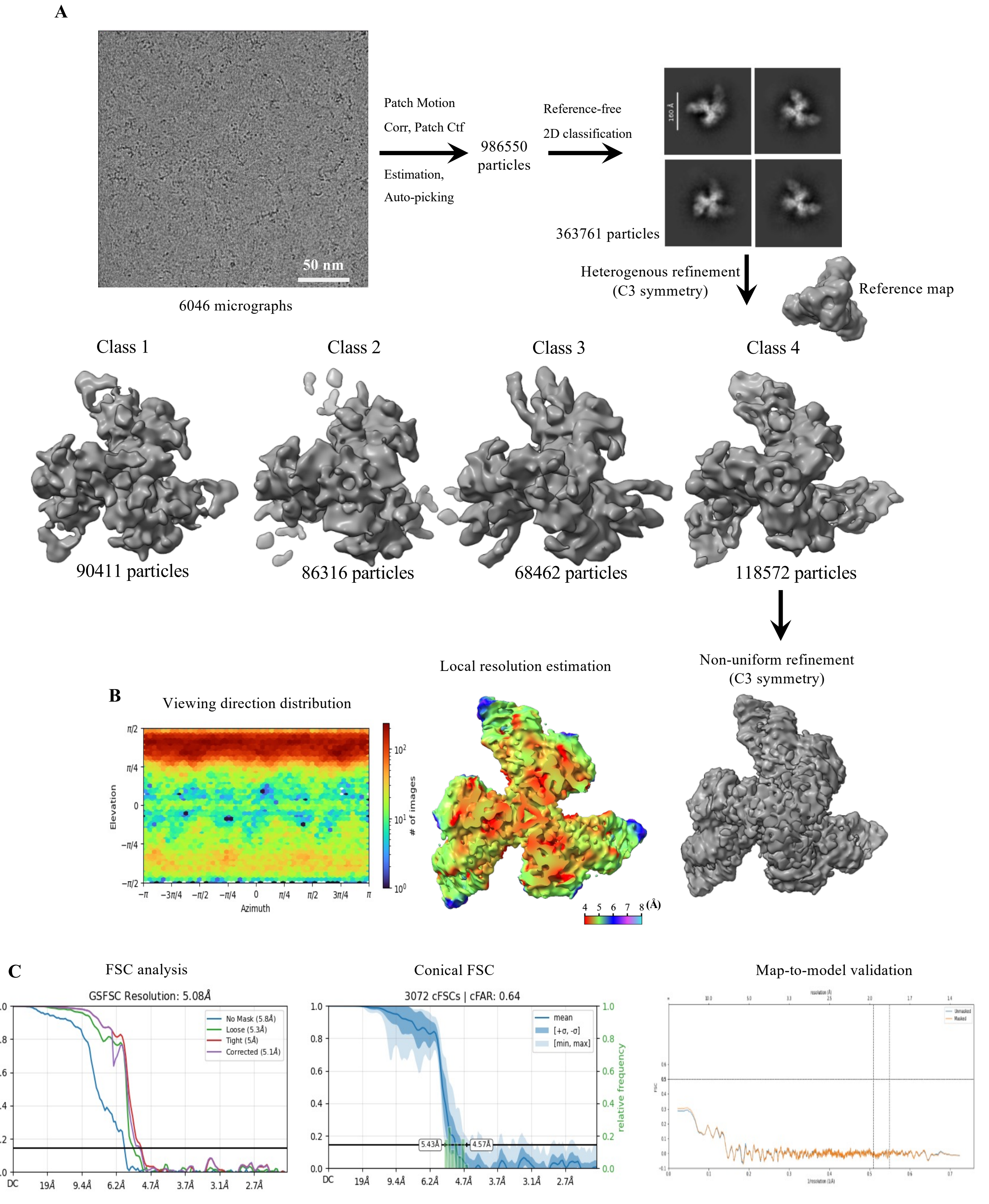

Figure S8: Cryo-EM analysis of 330 SOSIP.664 Env trimer complexed with autologous bnAb 44m. (A) Pipeline of Cryo-EM data processing using single particle cryo-EM and 3D classification of 330 SOSIP.664 Env trimer in complex with 44m bnAb. Ab initio reconstruction showed that the Class 04 map has better features with bnAb, and was therefore further considered for refinement and sharpening. **(B)** The viewing-direction particle distribution for Class 04 reveals diverse particle orientations in the dataset. **(C)** Gold standard Fourier Shell Correlation (FSC) curve of the cryo-EM map at 5.08 Å of 330 SOSIP Env trimer in complex with 44m bnAb.

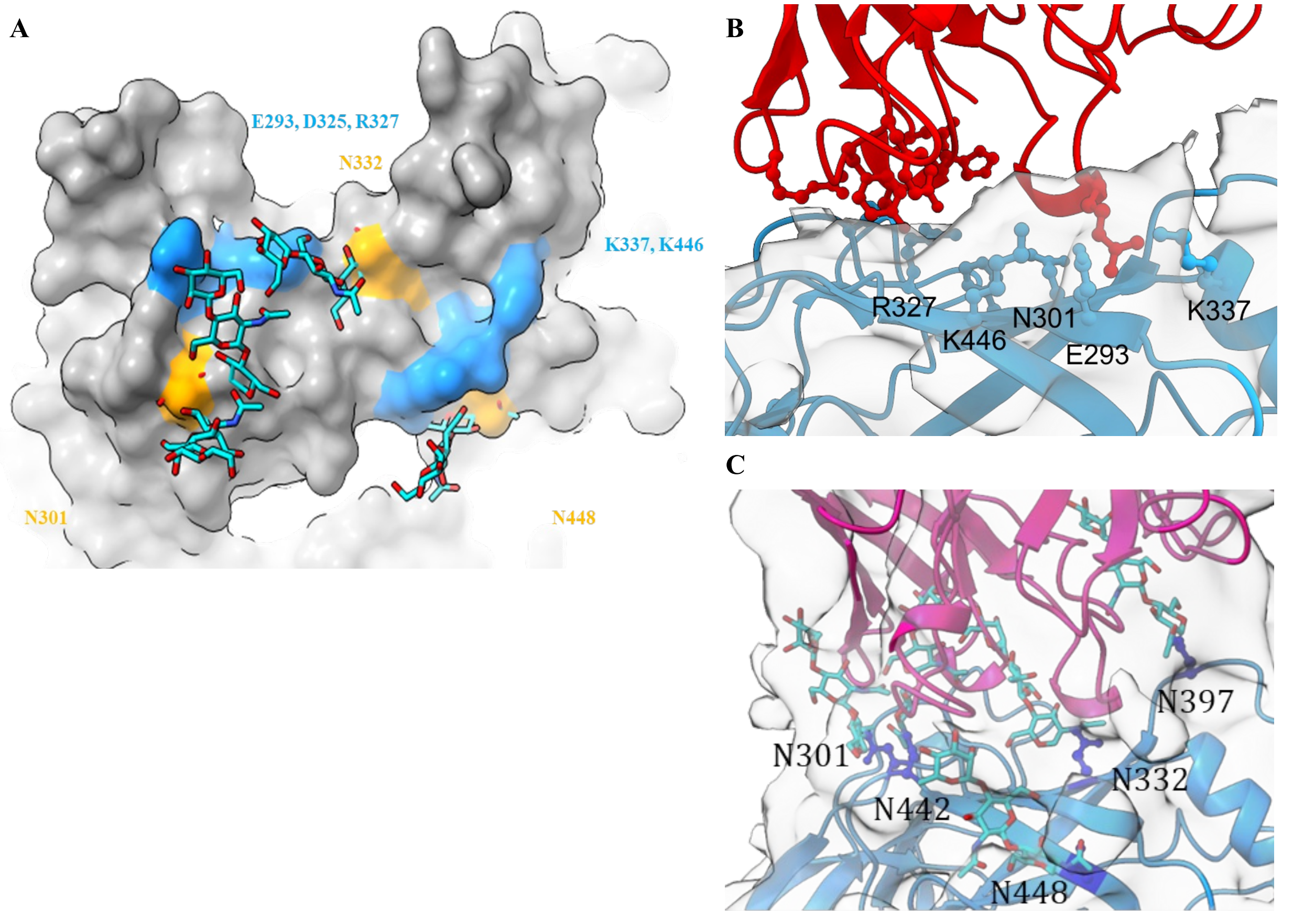

Figure S9: Structural interface analysis of the autologous Env 330 SOSIP.664 in complex with V3-directed bnAb 44m (A) Surface representation of gp120 residues and glycans involved in interactions with 44m. Contacting protein atoms are shown in blue, contacting glycan atoms in orange, and non-interacting regions in gray. **(B)** Representative EM density showing the fitting of interface amino acid residues and highlighting their contribution to the buried surface area analysis. **(C)** Fitting of Env glycans into the EM density map, supporting the buried surface area analysis

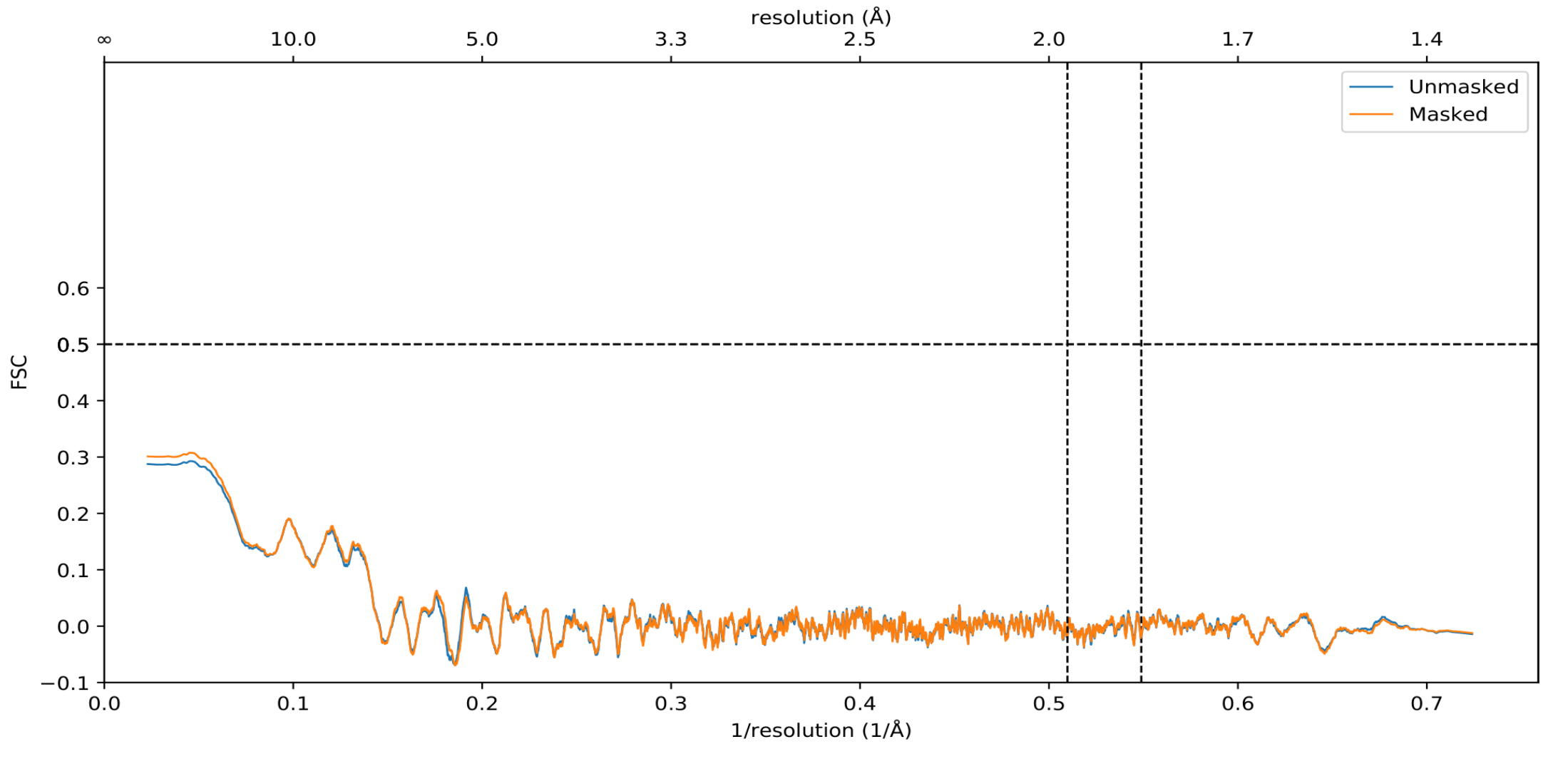

**Validation**	
MolProbity Score	2.52
Clash Score	17.36
Rotamer Outliers (%)	3.93
Ramachandran Plot	
Outlier (%)	0.09
Allowed (%)	4.59
Favored (%)	95.32

Figure S10: Cryo-EM map-to-model validation of the AIIMS 330 Env-44m bnAb complex. The AlphaFold-predicted models of AIIMS 330 Env and the autologous 44m bnAb were fitted into the 5.1 Å cryo-EM density map using ChimeraX and Coot. The fitted model was subsequently used for atomic model building and real-space refinement in Phenix 1.19.2. Map-to-model Fourier shell correlation (FSC) analysis was performed to assess the agreement between the refined atomic model and the cryo-EM density map. Refinement and model-validation statistics are summarized in **Table S8**.

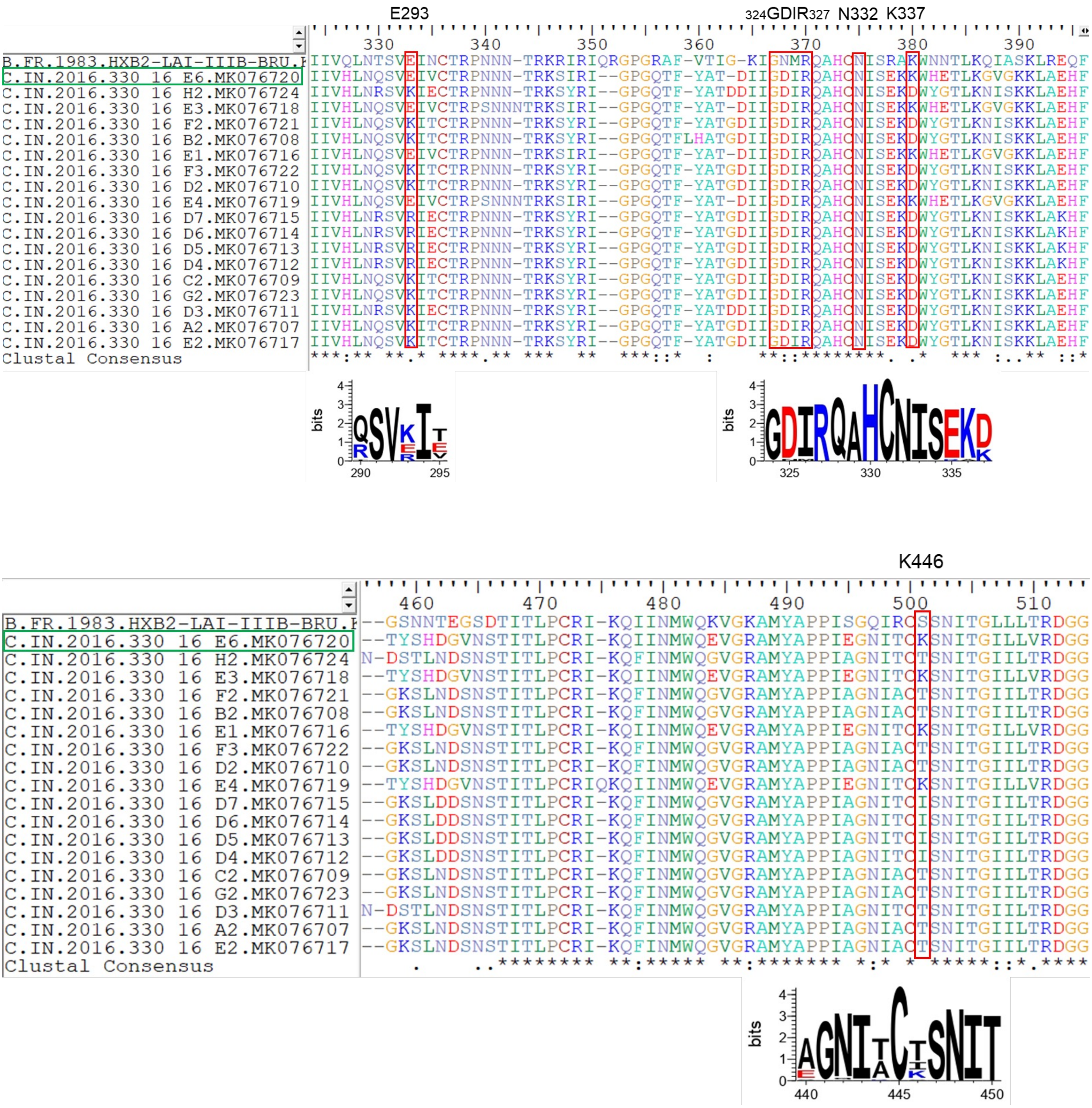

Figure S11: Sequence alignment analysis of Env variants. Sequence alignment of 18 HIV-1 Env variants generated from donor AIIMS 330 by single-genome amplification (SGA) from the previous study. The 330.E6.16 Env, used for immunogen design and structural characterization in complex with 44m by cryo-EM, is highlighted in green. Conserved V3-glycan supersite elements, including the N332 glycan and GDIR motif, are shared across all Env variants, whereas additional residues E293, K337, and K446 identified at the 330 Env-44m interface are restricted to E6, E1, E4, and E3 Env variants. Sequence conservation analysis and amino acid frequency at selected positions are highlighted using WebLogo.

Table S1. Envelope trimer-stabilizing mutations were introduced into the 330 SOSIP and NFL constructs.

Stabilizing Mutations	330_SOSIP	330_NFL	Role in stabilization
[1]Δ MPER	+	+	Soluble prefusion conformation
[1]R6 Furin site	+	-	
[1]I559P (IP)	+	+	
[1]501C-605C (SOS)	+	-	
[2](GGGGS)^{*2} linker	-	+	
[3]65K	+	+	Reduced exposure for V3 non-nAbs
[3]535M	+	-	
[3]543N	+	-	
[4]201C and 433C	+	+	Prevent sCD4 exposure
[5]47D	+	+	BG505 Trimer Derived (TD8) stabilizing mutations
[5]49E	+	+	
[5]65K	+	+	
[5]106T	+	+	
[5]165L	+	+	
[5]429R	+	+	
[5]432Q	+	+	
[5]500R	+	+	
[6]171K	+	+	V2 Apex optimization
[7]519S/R	S	R	Increased expression and trimerization
[7]520L/R	L	R	
[7]568D/G	D	G	
[7]569G	-	+	
[7]570H	+	-	
[7]585H	+	-	

[1] described in Sanders et. al. | 2013, PLoS Pathogens

[2] described in Sharma et. al. | 2015, Cell Reports

[3] described in Taeye et al., | 2015 | Cell

[4]described in Guenega et al. | 2017 | Immunity

[5]described in Guenega et al. | 2016 | JVI

[6]described in Andrabi et al. | 2015 | Immunity

[7]described in Steichen et al. | 2016 | Immunity

Table S2. Binding kinetics of 330 Env Variants Assessed via Biolayer Interferometry. A summarized table outlines the equilibrium dissociation constant (KD), association constant (Ka), and dissociation constant (Kdis) for bnAbs & non-nAbs tested with 330 gp140 envelope trimers (SOSIP & NFL) and ferritin NP's. The KD values were derived using 1:1 global fitting.

NB- No Binding

mAbs	KD (nM)	KD Error	ka (1/Ms)	ka Error	kdis (1/s)	kdis Error	Full X^2	Full R^2
PGT145	1.070E-09	1.16E-11	9.520E05	6.325E03	1.019E-03	8.756E-06	30.4837	0.7208
PGDM1400	1.344E-09	1.58E-11	4.031E05	1.638E03	5.417E-04	5.997E-06	17.0672	0.9131
PGT151	5.009E-12	-	6.775E04	1.261E02	3.393E-07	-	7.6675	0.9914
F105	7.158E-06	6.31E-05	4.048E02	3.568E03	2.897E-03	2.624E-04	17.3859	-
17b+sCD4	NB	-	7.747E04	4.022E03	2.441E-07	-	6.2921	0.3134
	**SOSIP.664**							

mAbs	KD (nM)	KD Error	ka (1/Ms)	ka Error	kdis (1/s)	kdis Error	Full X^2	Full R^2
PGT145	1.39E-09	7.87E-12	4.344E05	1.854E03	6.045E-04	2.243E-06	15.3614	0.9917
PGDM1400	3.32E-09	2.58E-11	1.783E05	9.826E02	5.926E-04	3.241E-06	7.1287	0.9874
PGT151	1.42E-11	-	1.777E04	1.362E03	2.520E-07	-	0.9021	0.7821
F105	2.35E-08	2.88E-10	7.727E04	8.742E02	1.812E-03	8.606E-06	0.5431	0.9632
17b+sCD4	NB	-	4.165E05	4.371E03	3.810E-07	-	0.1097	0.9624
	**NFL.664**							

mAbs	KD (nM)	KD Error	ka (1/Ms)	ka Error	kdis (1/s)	kdis Error	Full X^2	Full R^2
PGT145	1.1E-09	7.93E-12	7.139E05	2.550E03	7.840E-04	4.920E-06	46.0383	0.9144
PGDM1400	1.39E-09	1.99E-11	3.030E05	1.164E03	4.211E-04	5.811E-06	24.6365	0.9285
PGT151	3.02E-12	-	6.651E04	1.488E02	2.010E-07	-	8.2643	0.9862
F105	6.99E-08	1.76E-09	3.144E04	6.405E02	2.198E-03	3.229E-05	2.1896	0.7236
17b+sCD4	NB	-	1.233E05	3.768E03	2.441E-07	-	1.3081	0.3051
	**Ferritin NP**							

Table S3: Comparison of potential N-linked glycosylation sites (PNGS) across cross-clade envelopes. An N indicates the presence of a PNGS, if NXT/S motif in a.a. sequence, whereas X should not be proline. BG505 & 330 Envs share the same total number of PNGS and the same number of NXT & NXS N-glycosylation sites. Created by the N-glycosite tool using the Los Alamos HIV Sequence Database

Envelope	PNGS	NXT	NXS	88	133	135	136	137	139	141	142	147	156	160	186	187	188	197	230	234	241	262	276	289	295	301
BG505	28	16	12	N	N	-	N	-	-	N	N	-	N	N	N	-	-	N	N	N	N	N	N	N	N	N
330	28	16	12	N	-	-	N	-	-	N	-	-	N	N	N	N	-	N	N	N	N	N	N	N	N	N
16055	28	17	11	N	-	-	N	-	N	N	-	-	N	N	N	-	-	N	N	N	N	N	N	N	N	N
C97ZA	30	20	10	N	N	-	N	N	-	N	-	-	N	N	N	N	-	N	N	N	N	N	N	N	N	N
42J41	29	21	8	N	-	-	N	-	-	N	-	-	N	N	N	N	-	N	N	N	N	N	N	N	N	N
Du422	30	19	11	N	-	N	N	-	-	N	-	N	N	N	N	-	-	N	N	N	N	N	N	N	N	N
ZM246F	29	18	11	N	-	-	N	-	-	N	-	-	N	N	N	N	N	N	N	N	N	N	N	N	N	N

Envelope	332	334	339	355	356	360	363	386	392	397	398	399	403	404	406	407	409	411	442	448	461	462	463	505	611	616	618	624	625	637
BG505	N	-	N	N	N	-	N	N	N	N	N	-	-	-	N	-	-	N	-	N	-	N	N	-	N	N	N	N	N	N
330	N	-	N	N	N	-	-	N	N	N	-	-	-	-	N	N	-	N	N	N	-	N	N	-	N	N	-	N	N	N
16055	N	-	N	-	N	N	-	N	N	N	-	N	-	N	N	-	N	-	N	N	-	-	N	-	N	N	-	N	N	N
C97ZA	N	-	N	-	N	-	N	N	N	N	-	-	-	-	N	-	-	N	N	N	-	-	N	N	N	N	-	N	N	N
42J41	-	N	N	N	N	-	-	N	N	N	-	-	N	N	N	-	-	N	N	N	-	N	N	-	N	N	N	N	-	N
Du422	N	-	N	-	N	-	-	N	N	N	-	-	-	-	N	N	-	-	N	N	N	-	N	-	N	N	-	N	N	N
ZM246F	N	-	N	-	N	-	-	N	N	N	-	-	-	-	N	N	-	N	N	N	N	-	N	-	N	N	-	N	N	N

Table S4. Mutant pseudovirus neutralization assay to assess residue dependence. Cryo-EM identified Env contact residues (E293, D325, R327, H330, N332, K337, and K446) were individually mutated by site-directed mutagenesis (alanine substitutions, except N332Q). Mutant pseudoviruses were tested for antibody neutralization using a TZM-bl reporter assay. IC_{50} values represent the mean of independent experiments.

Mutant Neutralization		
	IC50 µg/mL	Fold Change
RTE6 WT	0.086	1
E293A	0.065	0.76
D325A	0.041	0.48
R327A	>10	116
H330A	0.092	1.07
N332Q	>10	116
K337A	0.055	0.64
K446A	<0.04	0.47

Table S5: Midpoint neutralization titers of rabbit immune sera at week 22 tested against a panel of Env-pseudotyped viruses. TZM-bl neutralization assays were performed in duplicate, and MLV (murine leukemia virus) was used as a negative control. The study is divided into four groups, Group 1, 2 & 3 (n=4) represent 330 NFL, SOSIP trimers & multivalent Ferritin NP's, while Group 4 (n=2) represents a placebo. The ID_{50} values, i.e., the serum dilution at which infectivity was inhibited by 50%, are shown and color-coded: white = no neutralization, ID_{50} < 20; yellow = weak neutralization, ID_{50} 20-100; orange = moderate neutralization, 100-300; red = strong neutralization, 300-2000. Individual ID_{50} value of immunized animal tested against env pseudotyped viruses.

			virus	MLV	330.16.E6	329.14.B1	MW965.26	25925	SF162	Bal01	BG505
			Tier	N/A	ND	ND	1A	1B	1A	1A	2
Study	Groups	Immunogen	Clade	control	C	C	C	C	B	B	A
LBPL/NG-1736 (EF)	Group 1	Ferritin NP	NZW5265	<20	1230	410	277	100	100	<20	<20
			NZW5266	<20	1450	530	255	77	122	<20	<20
			NZW5267	<20	1090	450	344	77	122	<20	<20
			NZW5268	<20	677	33	211	33	63	<20	<20
	Group 2	SOSIP	NZW5269	<20	890	470	100	33	55	<20	<20
			NZW5270	<20	830	473	166	<20	100	32	<20
			NZW5271	<20	710	450	144	100	144	<20	<20
			NZW5272	<20	830	270	144	77	77	<20	<20
	Group 3	NFL	NZW5273	<20	<20	<20	<20	<20	<20	<20	<20
			NZW5274	<20	155	60	36	90	<20	<20	<20
			NZW5275	<20	375	232	73	96	25	<20	<20
			NZW5276	<20	429	393	41	65	<20	<20	<20
	Group 4	Placebo	NZW5277	<20	<20	<20	<20	<20	<20	<20	<20

ID50
<20
20-100
100-300
300-2000

Table S6: BLI-Octet tandem competition matrix. Heatmap illustrating competitive binding between immune sera and reference HIV-1 bnAbs and non-neutralizing antibodies. A 35% competition threshold was used to define competing interactions.

Competition Matrix

Immune Sera	Ab#	PGT145	PGDM1400	VRC01	44m	10.1074	PGT121	F105	447-52d	412d	17b	PGT151
SOSIP	Placebo W0	0.1231	0.1192	0.0337	0.1703	0.1535	0.1428	0.1042	0.1623	0.0755	0.0894	0.3663
SOSIP	Placebo W22	0.1992	0.2432	0.0897	0.3465	0.2895	0.2018	0.1569	0.1832	0.1295	0.1102	0.2986
SOSIP	Ferritin NP W0	0.2388	0.2814	0.1244	0.3688	0.3207	0.2330	0.2160	0.2632	0.2000	0.1632	0.3601
SOSIP	Ferritin NP W22	0.0306	0.0465	0.0109	-0.0155	0.0342	0.0768	0.0845	0.1714	0.1281	0.0783	0.2296
SOSIP	SOSIP W0	0.1697	0.1869	0.0683	0.1748	0.1919	0.1643	0.1664	0.2584	0.1908	0.1199	0.3947
SOSIP	SOSIP W22	-0.0597	-0.0358	-0.0792	-0.0955	-0.0344	-0.0352	0.0443	0.1607	0.0977	0.0384	0.2173
NFL	Placebo W0		0.1114	0.0013	0.1198	0.1615	0.1693	0.1666	0.2377	0.2341	0.1860	0.2986
NFL	Placebo W22	0.1609	0.1794	0.0770	0.2573	0.2952	0.1830	0.1891	0.2603	0.2452	0.1684	0.3663
NFL	NFLW0	0.1966	0.2352	0.1292	0.3063	0.3546	0.2270	0.0813	0.1318	0.1224	0.0735	0.2093
NFL	NFLW22	-0.1333	-0.1297	-0.1213	-0.1239	-0.0343	0.0045	-0.0157	0.0660	0.0250	-0.0396	0.2296

Table S7: Cryo-EM data collection and processing of unliganded 330 SOSIP.664 Env trimer.

**Data collection and processing**	
Magnification	42000
Voltage (kV)	200
Electron exposure (e–/Å2)	60
Number of frames	20
Defocus range (μm)	-0.75 to –2.25
Pixel size (Å)	1.17
Symmetry imposed	C3
Number of movies	4353
Number of particles in model	89209
Map resolution (Å)	7.43
FSC threshold	0.143

Table S8: Cryo-EM data collection and processing of Env 330-44m bnAb complex.

**Data collection and processing**	
Magnification	42000
Voltage (kV)	200
Electron exposure (e–/Å2)	60
Number of frames	20
Defocus range (μm)	-0.75 to –2.25
Pixel size (Å)	1.17
Symmetry imposed	C3
Number of movies	6046
Number of particles in model	118572
Map resolution (Å)	5.1
FSC threshold	0.143
**Validation**	
MolProbity Score	2.52
Clash Score	17.36
Rotamer Outliers (%)	3.93
Ramachandran Plot	
Outlier (%)	0.09
Allowed (%)	4.59
Favored (%)	95.32

Table S9: Comparison of rabbit serum autologous neutralization titers elicited by different Env SOSIP trimers. Neutralization titers are presented as reciprocal serum dilutions corresponding to 50% inhibition ID_{50} of pseudoviruses expressing autologous Envs in TZM-bl cells.

	SOSIP.664 Env trimers								
Envelope	**330**	**329**	**ZM197**	**IPGE-THIVC**	**AMC009**	**B41**	**AMC008**	**BG505 (v4.2)**	**BG505 (v5.2)**
Subtypes	C	C	C	C	B	B	B	A	A
Origin	Pediatric	Pediatric	Adult	Adult		Adult	Adult	Pediatric	Pediatric
Categorization	Elite neutralizer	Elite neutralizer	Elite neutralizer	Elite neutralizer	Elite neutralizer	Elite neutralizer	Elite neutralizer	Founder virus	Founder virus
Disease Stage	Late Stage	Late Stage	Late Stage	Late Stage	Early Stage	Late Stage	Early Stage	Early Stage	Early Stage
Animal No.	Neutralization Titers (ID_{50})								
1	890	38	1010	2284	<20	249	219	1190	>4860
2	830	58	153	431	<20	965	299	2022	2554
3	710	50	64	104	<20	111	262	627	50
4	830	58	45	342	<20	601	272	1556	<20
5	–	–	114	–	48	<20	178	738	494
Average	815	51	277	790	48	481.5	246	1226.6	1990

References	Current study	S. Kumar et al | vaccines	Torrents et al. | JVI	R. Kumar et al. | PLOS pathogens	Schorcht et al. | JVI	de Taeye et al.| Cell	Torrents et al. | JVI	de Taeye et al.| Cell	Torrents et al. | JVI

Table S10. Longitudinal Binding Kinetics, Durability, and Boosting Profiles across Autologous and Heterologous Viral Strains. Data values represent mean binding levels ± standard deviation (SD). *P < 0.05, **P < 0.01 indicate statistically significant differences in cross-sectional post-hoc Tukey HSD pairwise multi-comparisons among the active immunogen variants. Global Linear Mixed-Effects Models (LMM) demonstrated highly significant main group effects, time effects, and group × time interactions across all tested viral populations (P < 0.001).

Virus / Strain	Immunogen Group	Baseline (W0)	Peak Boost 1 (W6)	Durability Trough (W20)	Recall Boost 2 (W22)	Statistical Comparisons (Tukey HSD Post-Hoc)
**330 (Autologous)**	330 Ferritin NP (n=4)	0.273 ± 0.026	1.539 ± 0.151	0.488 ± 0.100	1.874 ± 0.054	Global LMM: Group, Time, Group×Time: all P < 0.001. W6: NP vs SOSIP (P=0.681), NP vs NFL (P=0.019)*, SOSIP vs NFL (P=0.048)* W20: NP vs SOSIP (P=0.035)*, NP vs NFL (P=0.041)*, SOSIP vs NFL (P=0.002)** W22: NP vs SOSIP (P=0.002)**, NP vs NFL (P=0.001)**, SOSIP vs NFL (P=0.314)
	330 SOSIP (n=4)	0.268 ± 0.096	1.453 ± 0.147	0.664 ± 0.158	1.497 ± 0.227	
	330 NFL (n=4)	0.315 ± 0.073	1.209 ± 0.059	0.251 ± 0.106	1.317 ± 0.076	
	Placebo (n=2)	0.030 ± 0.002	0.070 ± 0.003	0.084 ± 0.007	0.066 ± 0.005	
**SF162 (Heterologous)**	330 Ferritin NP (n=4)	0.091 ± 0.067	1.428 ± 0.104	0.096 ± 0.029	1.669 ± 0.042	Global LMM: Group, Time, Group×Time: all P < 0.001. W6: NP vs SOSIP (P=0.851), NP vs NFL (P=0.900), SOSIP vs NFL (P=0.627) W20: NP vs SOSIP (P=0.278), NP vs NFL (P=0.900), SOSIP vs NFL (P=0.188) W22: NP vs SOSIP (P=0.677), NP vs NFL (P=0.900), SOSIP vs NFL (P=0.746)
	330 SOSIP (n=4)	0.062 ± 0.062	1.477 ± 0.119	0.422 ± 0.485	1.496 ± 0.499	
	330 NFL (n=4)	0.039 ± 0.020	1.390 ± 0.164	0.040 ± 0.028	1.644 ± 0.035	
	Placebo (n=2)	0.030 ± 0.002	0.070 ± 0.003	0.084 ± 0.007	0.066 ± 0.005	
**6240 (Heterologous)**	330 Ferritin NP (n=4)	0.079 ± 0.052	1.524 ± 0.085	0.068 ± 0.025	1.640 ± 0.071	Global LMM: Group, Time, Group×Time: all P < 0.001. W6: NP vs SOSIP (P=0.015)*, NP vs NFL (P=0.210), SOSIP vs NFL (P=0.248) W20: NP vs SOSIP (P=0.239), NP vs NFL (P=0.655), SOSIP vs NFL (P=0.063) W22: NP vs SOSIP (P=0.112), NP vs NFL (P=0.294), SOSIP vs NFL (P=0.774)
	330 SOSIP (n=4)	0.043 ± 0.030	1.334 ± 0.050	0.106 ± 0.041	1.534 ± 0.048	
	330 NFL (n=4)	0.037 ± 0.018	1.425 ± 0.085	0.048 ± 0.023	1.565 ± 0.074	
	Placebo (n=2)	0.030 ± 0.002	0.070 ± 0.003	0.079 ± 0.001	0.056 ± 0.005	
**1086 (Heterologous)**	330 Ferritin NP (n=4)	0.072 ± 0.052	1.067 ± 0.104	0.079 ± 0.020	1.271 ± 0.195	Global LMM: Group, Time, Group×Time: all P < 0.001. W6: NP vs SOSIP (P=0.212), NP vs NFL (P=0.002)**, SOSIP vs NFL (P=0.026)* W20: NP vs SOSIP (P=0.494), NP vs NFL (P=0.758), SOSIP vs NFL (P=0.200) W22: NP vs SOSIP (P=0.844), NP vs NFL (P=0.050)*, SOSIP vs NFL (P=0.117)
	330 SOSIP (n=4)	0.068 ± 0.029	0.091 ± 0.174	0.148 ± 0.143	1.225 ± 0.054	
	330 NFL (n=4)	0.029 ± 0.013	0.646 ± 0.027	0.038 ± 0.009	1.037 ± 0.037	
	Placebo (n=2)	0.030 ± 0.002	0.070 ± 0.003	0.084 ± 0.007	0.066 ± 0.005	
**329 (Heterologous)**	330 Ferritin NP (n=4)	0.051 ± 0.016	1.456 ± 0.055	0.062 ± 0.026	1.458 ± 0.115	Global LMM: Group, Time, Group×Time: all P < 0.001. W6: NP vs SOSIP (P=0.658), NP vs NFL (P=0.784), SOSIP vs NFL (P=0.900) W20: NP vs SOSIP (P=0.383), NP vs NFL (P=0.900), SOSIP vs NFL (P=0.420) W22: NP vs SOSIP (P=0.031)*, NP vs NFL (P=0.087), SOSIP vs NFL (P=0.775)
	330 SOSIP (n=4)	0.099 ± 0.064	1.513 ± 0.138	0.223 ± 0.281	1.672 ± 0.107	
	330 NFL (n=4)	0.064 ± 0.015	1.498 ± 0.057	0.071 ± 0.014	1.626 ± 0.062	
	Placebo (n=2)	0.059 ± 0.015	0.082 ± 0.010	0.149 ± 0.012	0.075 ± 0.004	
